# Gene Expression of Desaturase (*FADS1* and *FADS2*) and Elongase (*ELOVL5*) Enzymes in Peripheral Blood: Association with Polyunsaturated Fatty Acid Levels and Atopic Eczema in 4-Year-Old Children

**DOI:** 10.1371/journal.pone.0078245

**Published:** 2013-10-22

**Authors:** Aida Maribel Chisaguano, Rosa Montes, Teresa Pérez-Berezo, Ana Isabel Castellote, Marcela Guerendiain, Mariona Bustamante, Eva Morales, Raquel García-Esteban, Jordi Sunyer, Àngels Franch, M. Carmen López-Sabater

**Affiliations:** 1 Department of Nutrition and Food Science, Faculty of Pharmacy, University of Barcelona, Barcelona, Spain; 2 CIBER Fisiopatología de la Obesidad y Nutrición (CIBERobn), Instituto de Salud Carlos III (ISCIII), Spanish Government, Madrid, Spain; 3 Departament of Physiology, Faculty of Pharmacy, University of Barcelona, Barcelona, Spain; 4 Center for Research in Environmental Epidemiology (CREAL), Barcelona, Spain; 5 Hospital del Mar Research Institute (IMIM), Barcelona, Spain; 6 CIBER Epidemiología y Salud Pública (CIBERESP), Instituto de Salud Carlos III (ISCIII), Spanish Government, Madrid, Spain; 7 Department of Experimental and Health Sciences, Pompeu Fabra University, Barcelona, Spain; 8 Center for Genomic Regulation (CRG), Barcelona, Spain; University of Milan, Italy

## Abstract

**Background:**

It is unknown if changes in the gene expression of the desaturase and elongase enzymes are associated with abnormal n-6 long chain polyunsaturated fatty acid (LC-PUFA) levels in children with atopic eczema (AE). We analyzed whether mRNA-expression of genes encoding key enzymes of LC-PUFA synthesis (*FADS1, FADS2* and *ELOVL5*) is associated with circulating LC-PUFA levels and risk of AE in 4-year-old children.

**Methods:**

AE (n=20) and non-AE (n=104) children participating in the Sabadell cohort within the INfancia y Medio Ambiente (INMA) Project were included in the present study. RT-PCR with TaqMan Low-Density Array cards was used to measure the mRNA-expression of *FADS1*, *FADS2* and *ELOVL5*. LC-PUFA levels were measured by fast gas chromatography in plasma phospholipids. The relationship of gene expression with LC-PUFA levels and enzyme activities was evaluated by Pearson’s rank correlation coefficient, and logistic regression models were used to study its association with risk of developing AE.

**Results:**

Children with AE had lower levels of several n-6 PUFA members, dihomo-γ-linolenic (DGLA) and arachidonic (AA) acids. mRNA-expression levels of *FADS1* and *2* strongly correlated with DGLA levels and with D6D activity. *FADS2* and *ELOVL5* mRNA-expression levels were significantly lower in AE than in non-AE children (-40.30% and -20.36%; respectively), but no differences were found for *FADS1*.

**Conclusions and Significance:**

Changes in the mRNA-expression levels of *FADS1* and *2* directly affect blood DGLA levels and D6D activity. This study suggests that lower mRNA-expressions of *FADS2* and *ELOVL5* are associated with higher risk of atopic eczema in young children.

## Introduction

Atopic eczema (AE) is a multifactorial skin disease occurring most frequently in early childhood. It is caused by a complex interplay between immunological, genetics, biochemical, psychological and environmental factors. AE currently affects 15 to 30% of children and 1 to 3% of adults in industrialized countries, with 85% of cases beginning before the age of five [1,2]. 

**Figure 1:**
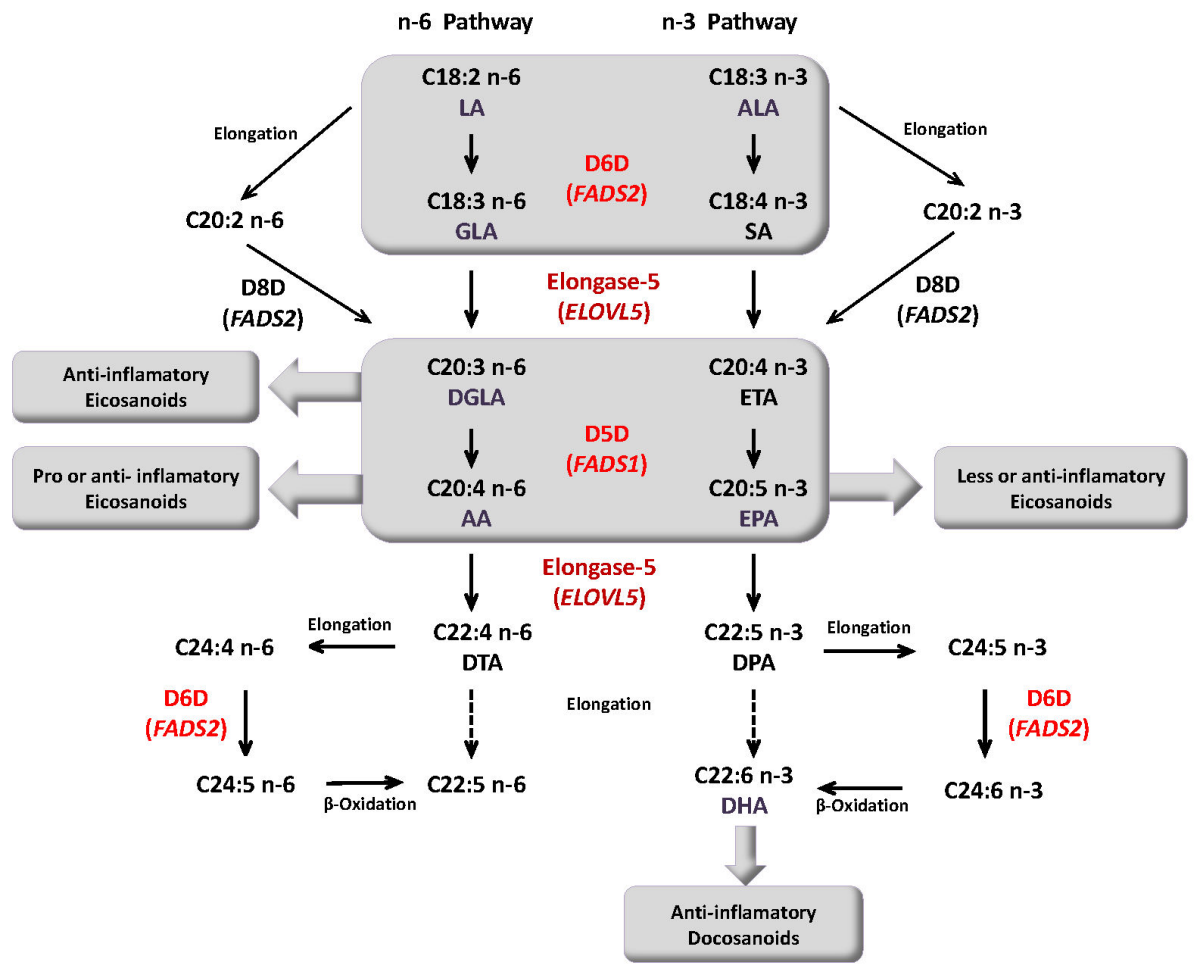
n-6 and n-3 pathways of long chain polyunsaturated fatty acid (LC-PUFA) biosynthesis from essential fatty acids (EFAs). Modified from [10,14,33]. LA, linoleic acid; GLA, γ- linolenic acid; DGLA, dihomo-γ-linolenic acid; AA, arachidonic acid; DTA, docosatetraenoic acid ALA, α-Linolenic acid; SA stearidonic acid; ETA eicosatetraenoic acid; EPA, eicosapentaenoic acid; DPA, docosapentaenoic acid; DHA, docosahexaenoic acid. D5D (Δ5-desaturase), D6D (Δ6-desaturase) and Elongase-5 enzymes are encoded by *FADS1, FADS2* and *ELOVL5*, respectively.

Many studies suggest that AE is associated with impairment in the metabolism of n-6 essential fatty acids (EFAs) and long chain polyunsaturated fatty acids (LC-PUFAs). Deficits in n-6 EFAs are related to the severity of AE through changes in skin barrier function and cutaneous inflammation [3-6]. Higher levels of linoleic acid (LA, C18:2 n-6) and significantly lower levels of γ- linolenic (GLA, C18:3 n-6), dihomo-γ-linolenic (DGLA, C20:3 n-6) and arachidonic (AA, C20:4 n-6) acids have been noted in the plasma or serum of AE children compared to healthy control [3,4,7,8]. The synthesis of LC-PUFAs from LA involves enzyme-mediated desaturation and elongation steps. Δ6-desaturase (D6D, encoded by the *FADS2* gene) catalyzes the conversion of LA into GLA, which is then elongated into DGLA by elongase-5 (encoded by the *ELOVL5* gene). Moreover, DGLA can be converted into AA by Δ5-desaturase (D5D, encoded by the *FADS1* gene) (summarized in Figure 1) [9-13].

According to findings of different studies, defects in the activity of enzymes involved in LC-PUFAs biosynthesis could be related to lower concentrations of LA metabolites [7,14]. Recent studies report an association between genetic variants (Single Nucleotide Polymorphism, SNP) of the *FADS1* and *FADS2* gene cluster and LC-PUFA levels in serum, plasma phospholipids and erythrocyte membranes. Subjects carrying the minor alleles have higher LA and DGLA levels, and lower AA concentrations [10,15-17]. Furthermore, a link between *FADS* SNPs and atopic outcomes in children, such as AE and asthma, has been investigated with controversial results. Rzehak et al.[14], using two population-based birth cohorts, found that PUFA composition in young children’s blood was strongly controlled by the *FADS* gene cluster. These authors reported that SNPs were significantly associated with eczema in the LISA, but not the KOALA cohort. On the other hand, Standl et al. [11] and Sigman et al. [18] concluded that there was no association between *FADS* polymorphisms and atopic diseases in children. 

To date, it is unclear whether changes in the expression of *FADS1* and *FADS2*, and consequently, LC-PUFA levels in children and adults determine risk of atopic diseases in humans. To our knowledge, only one study developed by Saaf et al. [19] reports a reduced gene expression of *FADS1* (D5D) and *FADS2* (D6D) in the AE skin of adults. Their results support the hypothesis that low LC-PUFA levels are due to impaired desaturase activity and thus, impaired synthesis of these fatty acids [7]. As far as we know, there are no scientific studies focusing on the mRNA-expression of these genes in children.

Combining measurements of gene expression, enzyme activity and polyunsaturated fatty acid levels is essential in explaining the differences in blood lipid levels of children who develop atopic diseases. Thus, the aim of this study was to investigate whether mRNA-expression of genes encoding key enzymes of LC-PUFA synthesis (*FADS1, FADS2* and *ELOVL5*) is associated with circulating LC-PUFA levels and risk of AE in 4-year-old children**.**

## Methods

### Ethics statement

Written informed consent was obtained from all participant parents and the study was approved by the Clinical Research Ethical Committee of the Municipal Institute of Health Care (CEIC-IMAS) and the Hospital of Sabadell, Spain.

### Study population and design

Data for this report come from a population-based pregnancy cohort set up in Sabadell, Spain as part of the INMA Project [Cohort Profile: The INMA--INfancia y Medio Ambiente--(Environment and Childhood) Project] [20]. A detailed description of the Sabadell cohort has been provided elsewhere [21,22]. A total of 657 children were enrolled between 2004 and 2006. At 4 years of age a subset of 124 children were included in the present study

During pregnancy and the first 4 years postpartum, information on maternal education, gender, breastfeeding and atopic outcomes was collected through questionnaires administered to the mothers at various time points (12 and 32 weeks of gestation, as well as follow-up at 6 and 14 months and 4 years postpartum). 

### Definition of eczema

Information about AE was obtained from the parents through two questionnaires completed when the child was 14 months [22] and 4 years old. Three questions were addressed to the parents: at 14 months old, “has your child ever experienced atopic eczema since birth to 14 months?” and at 4 years, “has the child ever experienced atopic eczema in the last 12 months?” and “has the child taken any medication for atopic eczema in the last 12 months?” AE was defined as recurrent atopic eczema (reported symptoms during the first year of life and at 4 years old) and having medication for eczema at age 4 (n=20). Non-AE had no personal history of AE (n=104).

### RNA isolation and cDNA synthesis

Venous peripheral blood samples obtained from 4-year-old children were collected in PAXgene (TM) Blood RNA tubes (BD company, Germany) to ensure RNA preservation and stored at -20 and then at -80C before RNA extraction. Total RNA was isolated from whole blood using the PAXgene Blood RNA Kit following the manufacturer’s protocol (Qiagen, Hilden, Germany). RNA concentration was determined using a spectrophotometer (Nanodrop) and quality (percentage of miRNAs and RIN) was determined by the Bioanalyzer system (Agilent, USA). 700 ng of total RNA was reverse-transcribed in the thermal cycler PTC-100 into cDNA using the SuperScript cDNA Synthesis Kit, according to the manufacturer’s protocol (Invitrogen, USA). The reaction mixture was incubated at 25°C for 10 min and then at 42°C for 60 min. Finally, the mixture was heated at 85°C for 5 min. cDNA samples were stored at -80°C prior to analysis.

### PCR using TaqMan low-density array cards (TLDAs)

To identify altered gene expression in peripheral blood, we used PCR with TaqMan Low-Density Array (TLDA) microfluidic cards (Applied Biosystems, USA). Each port was preloaded with specific user-defined TaqMan primers and probes. This technology has been shown to be sensitive and reproducible for assessing gene expression [23,24]. Although the TLDA cards were predesigned to measure 24 genes, we only used it for the genes related to the elongation and desaturation of LC-PUFAs. Thus, each card contained the target genes *ELOVL5* [Hs 01094704_m1, inventoried], *FADS1* [Hs 00203685_m1, inventoried] and *FADS2* [Hs 00188654_m1, inventoried]. Additionally, each card contained five endogenous control genes, *PPIB* [Hs 00168719_m1, inventoried], *FPGS* [Hs 00191956_m1, inventoried], *TRAP1* [Hs00212474_m1, inventoried], *GAPDH* [Hs99999905_m1 inventoried] and *β2M* [Hs99999907_m1, inventoried]. The full RT-PCR data set is available at The NCBI Gene Expression Omnibus (GEO) Database (GEO accession: GSE48310)

A total of 100 μL of reaction mixture, with 16 μL of cDNA (560 ng), 34 µL of H_2_O and 50 μL of TaqMan Universal PCR Master Mix (Applied Biosystems, USA), was added to each TLDA port after vortexing. The card was centrifuged for 1 min x 2 at 1900 rpm to distribute the samples from the loading port to each well. The TLDA was sealed with a TaqMan low-density array sealer (Applied Biosystems, USA) and the PCR amplification was performed using an ABI Prism 7900HT sequence detection system (Applied Biosystems, USA). The thermal cycler conditions were as follows: 2 min at 50°C, then 10 min at 94.5°C, followed by 40 cycles for 30 s at 97°C and 1 min at 59.7°C. Each sample was measured in duplicate in the same TLDA card. The SDS software (version 2.4) was used to analyze the expression data.

The amount of target mRNA relative to the endogenous control expression and relative to values from the reference group was calculated using the 2^−ΔΔC^_t_ method, as previously described [25]. mRNA-expression levels were normalized using the expression of peptidylpropyl isomerase B (*PPIB*) as the endogenous housekeeping gene, since this gene presented the highest stability in all the samples. Results were expressed as the mean of the percentage of these values for the experimental group (AE children) compared to the reference group (non-AE children), which represented 100% gene expression.

### Plasma phospholipid fatty acid analysis

The fatty acid composition of plasma phospholipids was determined according to the method described by Chisaguano et al. [26]. Fatty acid methyl esters (FAMEs) were prepared with sodium methylate in 0.5M methanol and boron trifluoride methanol solution (14% v/v). They were then separated and quantified by fast gas chromatography with flame ionization detection (FID). FAMEs were identified by comparing the peak retention times of the FAME samples with those of the standard FAME mix solutions. Data acquisition and processing were performed with the Shimadzu-Chemstation software for GC systems. The relative amount of each fatty acid quantified was expressed as the percentage of the total amount of fatty acids (% total fatty acids).

Enzyme activities were calculated using relevant fatty acid product:precursor ratios, as previously described [16,21,27]. Thus, the ratios C20:4 n-6/C20:3 n-6 (AA:DGLA) for D5D, C20:3 n-6/C18:2 n-6 (DGLA:LA) for D6D; C20:3 n-6/C18:3 n-6 (DGLA:GLA) and C22:4 n-6/C20:4 n-6 (DTA:AA) for Elongase-5 were used .

### Statistical analysis

Means and standard deviations (SDs) were used to describe continuous variables. The Kolmogorov-Smirnov test was used to study the normal distribution of data and non-normally distributed data were naturally log-transformed. The differences in gene expression between the groups were evaluated by unpaired Student t-test. Correlations between fatty acids, enzyme activities and mRNA-expression levels were determined by Pearson’s rank correlation coefficient, while logistic regression was applied to evaluate the association between mRNA levels and AE. The confounding variables were selected based on previous literature data and were gender, maternal education and breastfeeding [14,21]. All statistical analyses were performed on SPSS 20.0 for Windows (SPSS Inc. Chicago, IL, USA). 

## Results

### Population characteristics, PUFA levels and enzyme activity

The basic population characteristics, n-6 and n-3 LC-PUFA levels of plasma phospholipids, as well as enzyme activities (determined as fatty acid ratios) are summarized in Table 1. No statistical differences were found between AE and non-AE children for sex, predominant breastfeeding and maternal education (p> 0.05). 

**Table 1:** Characteristics of the Sabadell cohort subsample composed of 4-year-old children.

**Characteristics**	**Control (n=104**)	**Atopic eczema (n=20)**	p*
Gender [% male]	49	60	0.369^a^
Predominant breastfeeding for at least 2 weeks [%]	77.7	70	0.460^a^
Maternal education^т^ [%]	77.7	66.7	0.313^a^
**^1^ Fatty acids (mean % of total FA) ^†^**			
Linoleic (LA, C18:2 n-6)	25.21 ± 2.26	25.84 ± 2.30	0.282^b^
γ-Linolenic (GLA, C18:3 n-6)	0.09 ± 0.04	0.09 ± 0.06	0.545^b^
Dihomo-γ-Linolenic (DGLA, C20:3 n-6)	2.72 ± 0.66	2.40 ± 0.42	0.014^b^
Arachidonic (AA, C20:4 n-6)	9.48 ± 2.45	8.46 ± 1.77	0.046^b^
Adrenic (DTA, C22:4 n-6)	0.38 ± 0.10	0.38 ± 0.11	0.920 ^b^
α-Linolenic (ALA, C18:3 n-3) ^‡^	0.10 ± 0.04	0.11 ± 0.04	0.423^b^
Eicosapentanoic (EPA, C20:5 n-3) ^‡^	0.31 ± 0.16	0.30 ± 0.39	0.723^b^
Docosapentaenoic n-3 (DPA, C22:5 n-3)	0.48 ± 0.13	0.46 ± 0.19	0.680^b^
Docosahexanoic (DHA, C22:6 n-3)	2.35 ± 0.90	2.29 ± 1.03	0.811^b^
^1^ **Enzyme activities (fatty acids ratios**)			
Elongase 5 (*ELOVL5*)			
DGLA:GLA	36.26 ± 17.10	34.14 ± 16.23	0.628^b^
DTA:AA^Ѣ^	4.09 ± 0.76	4.49 ± 0.79	0.049^b^
D5D activity (*FADS1*)			
AA:DGLA	3.57 ± 0.91	3.58 ± 0.82	0.962^b^
D6D activity (*FADS2*)			
DGLA:LA^Ѣ^	10.96 ± 3.13	9.35 ± 1.61	0.002^b^

^т^ Secondary and university. ^1^ Fatty acids and enzyme activities (control, n= 94; atopic eczema, n=18). ^†^ Mean±SD. ^‡^ Natural log- transformation. ^Ѣ^ Fatty acid ratios x 100. ^a^ Significance for Pearson’s chi-squared test. ^b^ Significance for Student t- test.*p<0.05.

AE children tended to have higher LA levels, but this was not statistically significant (p= 0.282). Moreover, DGLA and AA levels were significantly lower in AE children than in controls (p= 0.014 and p= 0.046, respectively) (Table 1). We observed no significant differences in the levels of n-3 LC-PUFAs (EPA, DPA and DHA) between the two groups. 

There were no significant differences in D5D activity (AA:DGLA, p= 0.962) between AE children and control. However, AE children displayed lower D6D activity (DGLA:LA, p= 0.002) than controls. On the other hand, there were significant differences in elongase-5 activity (DTA:AA, p=0.049) between the two groups. 

### Relationship between gene expression and fatty acid levels

There was a positive relationship between the mRNA-expression levels of *FADS1* and *FADS2* (r=0.527, p=4.37E-10), suggesting that both genes are coexpressed. *FADS2* and *FADS1* expression positively correlated with DGLA and GLA levels and with the DGLA:LA ratio. 

In contrast, no correlation was found between *ELOVL5* (gene not located in the FADS cluster) and *FADS1* or *FADS2* mRNA-expressions. In addition, there was no observed correlation between *ELOVL5* gene expression and LC-PUFA levels or enzyme activities (Table 2).

**Table 2:** Relationship between the mRNA-expression level of each enzyme and enzyme activity in the peripheral blood of children.

			**Peripheral whole blood mRNA expression**			
	*FADS1*			*FADS2* ^‡^		*ELOVL5*
*FADS1*				0.527	**	
*ELOVL5*	0.160			0.078		
GLA	0.194	*		0.179		- 0.003
DGLA	0.255	**		0.332	**	0.031
AA	- 0.073			- 0.172		0.027
**Enzyme activity**						
Δ5-desaturase (*FADS1*)						
AA:DGLA	- 0.342	**		- 0.480	**	- 0.082
Δ6-desaturase (*FADS2*)						
DGLA:LA	0.218	*		0.328	**	0.014
Elongase-5 (*ELOVL5*)						
DGLA:GLA	-0.081			0.030		0.014
DTA:AA	- 0.038			0.170		0.088

^‡^ Natural log- transformation. Associations are presented as Pearson’s rank correlation coefficients. *p<0.05. ** p<0.01.

### Association between activity/mRNA-expression of enzymes and atopic eczema

**Figure 2:**
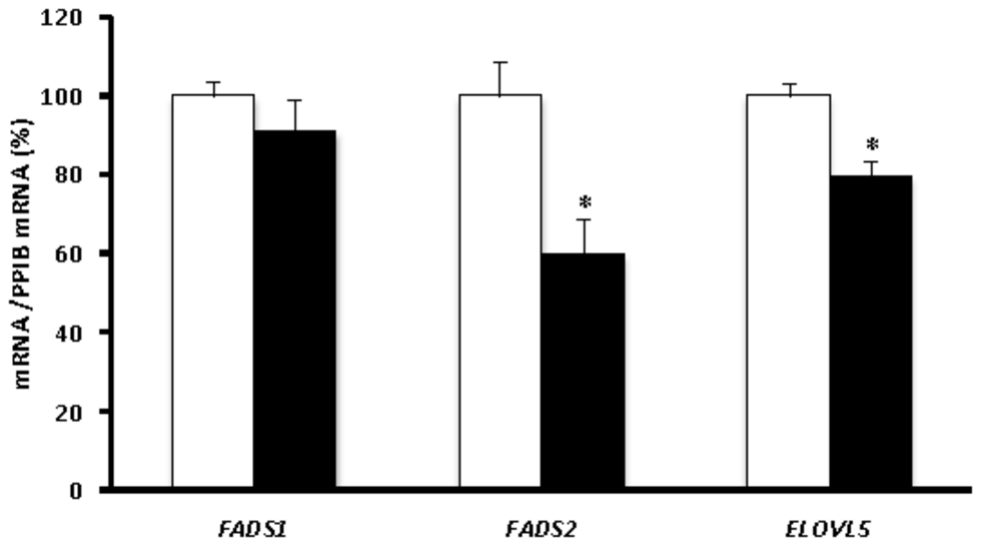
Fatty acid desaturase and elongase mRNA-expression (mRNA/PPIB mRNA) in peripheral blood. Expression levels were normalized using the expression of *PPIB* as the endogenous housekeeping gene. Black bars correspond to the AE children (n=20), and white bars correspond to the non-AE children (n=104). *FADS1* encodes Δ5-desaturase (-9.40%, D5D), *FADS2* Δ6-desaturase (-40.30%, D6D) and *ELOVL5* elongase-5 (-20.36%). Each bar represents the mean ± S.E.M. of the percentage of gene expression in AE children compared to the reference group, which showed 100% expression. *p<0.05.

Reduced mRNA-expression of the *ELOVL5* (-20.36%, p=0.005) and *FADS2* (-40.30%, p=0.013) genes was observed in the peripheral blood of AE children relative to controls. *FADS1* mRNA-expression was detected in AE children, but its level was not significantly different from that of control (-9.40%, p=0.308) (Figure 2).

Table 3 gives the results of the regression logistic models. There was no significant association between D5D activity (AA:DGLA) [OR=1.11, IC-95 (0.60-2.05), p=0.750] and atopic eczema. D6D activity (DGLA:LA) showed a marginal association with atopic eczema [OR=0.82, IC-95 (0.66-1.00), p=0.057]. On the other hand, elongase-5 activity determined by DTA:AA ratio showed significant a association with atopic eczema [OR=2.32, IC-95 (1.09-4.92), p=0.029].

**Table 3:** Association between enzyme activities, mRNA expression of each enzyme and atopic eczema.

	**N (AE/control)**	**OR unadjusted**	**95%-CI**	**p-value**	**N (AE/control)**	**OR adjusted^1^**	**95%-CI**	**p-value**
**Enzyme activities**								
D5D (AA/DGLA)	18/94	1.01	0.58 - 1.79	0.962	16/92	1.11	0.60 - 2.05	0.750
D6D(DGLA/LA)	18/94	0.82	0.67 - 0.99	0.041	16/92	0.82	0.66 - 1.00	0.057
ELOVL5 (DGLA/GLA)	18/93	0.63	0.20 - 2.04	0.444	16/91	0.98	0.26 - 3.64	0.970
ELOVL5 (DTA/AA)	18/94	1.90	0.99 - 3.64	0.054	16/92	2.32	1.09 - 4.92	0.029
**mRNA expression**								
*FADS1* (D5D)	20/104	0.99	0.98 - 1.01	0.306	18/99	0.99	0.97 - 1.00	0.089
*FADS2* (D6D)	19/103	0.42	0.17 - 1.04	0.061	17/98	0.20	0.05 - 0.74	0.017
*ELOVL5* (Elongase-5)	20/104	0.97	0.97 - 0.99	0.006	18/99	0.97	0.95 - 0.99	0.016

Odd ratios (OR) of measured mRNA expression and enzyme activities on eczema atopic are estimated by logistic regression. **^1^**Adjusted for maternal education, sex and predominant breastfeeding (> 2 weeks).

There was a significant association between *FADS2* [OR=0.20, IC-95 (0.05-0.74), p=0.017] or *ELOV5* [OR=0.97, IC-95 (0.95-0.99), p=0.016] mRNA-expression and the prevalence of AE in young children. Furthermore, a positive, but not significant, trend was found for *FADS1* [OR=0.99, IC-95 (0.97-1.00), p=0.089]. 

## Discussion

This is the first study to explore the gene expression of enzymes involved in LC-PUFA biosynthesis in whole peripheral blood, its relationship with enzyme activities and association with AE in 4-year-old children. We studied a subsample of children who developed AE in their first year of life (16.1% of total samples analyzed n=124). 

First, differences in LC-PUFA levels between AE and non-AE children were examined. It is known that the skin is the target organ in patients with atopic eczema; however, previous studies suggested that the GLA, DGLA and AA present in epidermal phospholipids are biosynthesized endogenously elsewhere and transported by the bloodstream to the epidermis, where phospholipid etherification takes place. Consequently, serum or plasma phospholipid levels of n-6 PUFAs may reflect their epidermal concentrations [4,28]. Our results confirmed that children with AE had lower DGLA levels in their blood than controls. DGLA is a direct precursor of the series-1 prostaglandins, such as PGE1. This prostaglandin has potent anti-inflammatory effects and is important for maintaining healthy skin by regulating water loss and protecting the skin from injury and infection [29-33]. Accordingly, we found that GLA was not effectively converted into DGLA in AE children, therefore indicating a reduction in the amounts and beneficial effects by action of PGE1. Furthermore, levels of AA, which is a direct precursor of anti- and pro-inflammatory mediators, were lower in AE children [3,34-36]. Our results suggest that an alteration in the endogenous biosynthesis of DGLA and AA leads to their decreased plasma levels. Regarding blood n-3 LC-PUFA levels, no changes were observed in the levels of ALA, EPA and DHA in AE children compared to controls. The differences in the associations of n-3 and n-6 LC-PUFAs with AE might be due to the fact that the former are more dependent on nutritional influences while the latter rely more on endogenous biosynthesis. These results are consistent with those reported by other authors in previous publications [37,38].

It has been hypothesized that the lower levels of n-6 LC-PUFAs in the serum, plasma or tissues of AE patients are related to defects in the D6D enzyme (encoded by *FADS2*) [3,7,39]. In this study, we observed decreased *FADS2* expression in AE children, in accordance with the results of Saaf et al. [19], who undertook genome-wide expression profiling in AE. Those authors analyzed the most important genes in lipid metabolism in the skin of adult AE patients (aged between 21 and 43 years), and found reduced *FADS1* and *2* expressions in AE patients relative to controls. In contrast to that study, we observed no significant differences in the expression levels of the *FADS1* gene; however, the results had a similar direction with Saaf (lower FASD1 in AE). Taken together, these findings suggest that the activity of local desaturases is predominantly localized in the skin of patients with severe AE, as seen with the activity of some markers of chronic inflammation and Th1 response, which are presented predominantly in the skin but not in the blood of AE patients [40]. Regarding elongase-5 expression, AE children had lower *ELOVL5* mRNA levels in their blood than their healthy counterparts. Therefore, these results could explain why AE children have lower levels of DGLA and AA, since D6D and elongase-5 directly participate in the biosynthesis of these fatty acids. 

Assessing the relationship between gene expression and fatty acid levels, we found that DGLA levels and D6D activity (DGLA:LA ratio) were directly associated with *FADS1* and *FADS2* gene expression. However, we did not find any relationship between activity (estimated by DTA:AA ratio) and mRNA-expression of elongase-5. This might be explained by the participation of other enzymes in the elongation of AA into DTA. Indeed, recent studies in several transgenic mouse models and cell cultures suggest that elongase-2 (encoded by *ELOVL2*) also participate in the elongation of AA [41]. Accordingly, in our study, AE and non-AE children displayed similar plasma levels of DTA, but different AA levels. We also measured elongase-5 activity using the DGLA:GLA ratio; however, there was no link between mRNA-expression and enzyme activity. In summary, the fatty acid ratios can be used to estimate D5D and D6D activities, but not that of elongase-5 in peripheral blood. 

Our results suggested that decreased *FADS2* and *ELOVL5* mRNA-expression in the blood could contribute to the development of AE in the first 4 years of life. Lower DGLA:LA ratios were associated with AE in an unadjusted multiple logistic regression model. When the regression was adjusted for the confounders (maternal education, predominant breastfeeding and sex), this relationship became non-significant as these confounders also modulated plasma fatty acids levels, as already demonstrated in previous studies [14,22]. Collectively, these data support the conclusion that the n-6 LC-PUFA deficits observed in 4-year-old AE children could be due to the reduced expression of the genes encoding Δ6-desaturase and elongase-5.

The main limitation of this study was the sample size because we analyzed only a subsample of the Sabadell cohort (in total, 657 neonates were recruited at birth between 2004 and 2006). However, for this study we selected only severe cases of AE. Thus, we consider the size of the subsample enough, since the cases of children affected with this skin disorder represented the 16.1 % of the total cases studied. Moreover, complementary studies regarding the impact of different genetic variants (FADS and ELOVL SNPs genotypes) on gene expression would be of great interest. Therefore, the results from our study need to be interpreted with caution and further replications in other cohorts are required to support our conclusions.

## Conclusion

This is the first study to confirm that *FADS2* and *ELOVL5* mRNA-expression is significantly lower in the peripheral blood of young children with atopic eczema. These changes in gene expression may explain the low concentration of n-6 LC-PUFAs in children who develop this skin disorder in their early childhood.
